# Trends and Patterns of Dengue Incidence and Mortality in Bangladesh: A Re‐Emerging Public Health Issue

**DOI:** 10.1002/puh2.70460

**Published:** 2026-11-07

**Authors:** Md. Idris Ali

**Affiliations:** ^1^ Department of Population Sciences University of Dhaka Dhaka Bangladesh

**Keywords:** Bangladesh, cases, deaths, dengue, outbreak, trend

## Abstract

**Background:**

Bangladesh faced a rapid dengue outbreak in its history after the year 2020 which marked as a re‐emerging health issue. This study aims to assess the significant change of the trends and patterns of dengue incidence and mortality over the year from 2000 to 2025 in Bangladesh.

**Methods:**

The publicly available government data were used from the Management Information System of the Ministry of Health and Family Welfare, Bangladesh, from 2000 to 2025. The data were cleaned, coded, and a descriptive analysis was performed using the Microsoft Excel. For assessing the significant trend of dengue cases, deaths, and case fatality rate (CFR), the Mann–Kendall test and Sen's slope estimator were conducted using the RStudio version 4.6.0 software. Moreover, Joinpoint regression software was used to analyze the temporal trends of dengue cases and deaths with *p* value less than 0.05 considered statistically significant.

**Results:**

According to the findings, the highest dengue record cases and deaths were reported in 2023 where 321,179 cases and 1705 deaths occurred as highest in the history of Bangladesh. Females and the elderly were most vulnerable in 2023 and other years as well. The CFR was high among the older population aged 61+, as highest in 2024. Most of the dengue cases and deaths occurred from July to December. The dengue cases and deaths significantly increased over the year from 2000 to 2025. But the CFR was not statistically significant over the year.

**Conclusion:**

Dengue significantly increased over the year and has become more fatal since 2020. More effective strategies are needed to prevent and control the exposure of cases and deaths of dengue. The policy suggests to enhancing the awareness programs for preventing dengue exposure and strengthening the health system capacity for government.

## Graphical abstract

Bangladesh faced a rapid dengue outbreak of cases and deaths in its history which marked as a re‐emerging health issue that significantly increased from 2000 to 2025. Older people and the Dhaka city population who are more vulnerable should be targeted by considering seasonal effect of dengue from July to December.

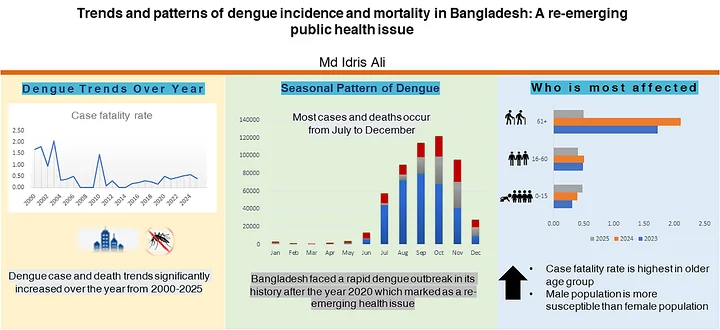

## 1 Introduction

Dengue is the fastest spreading viral infection and mosquito‐borne infectious disease caused by the dengue virus (DENV), which has re‐emerged as a public health problem globally [1]. Around 14.1 million dengue cases were reported, and 9508 deaths occurred related to dengue in 2024, in which half of the world population is at risk of dengue globally [1, 2]. According to World Health Organization (WHO), more than 4 million cases and 3000 deaths were reported from 97 countries to WHO from January to July 2025 [1]. The transmission of dengue has occurred due to many factors, such as population density, climate change, internal and international migration, urbanization (especially unplanned urbanization), and community risk factors like knowledge, attitude, and practices (KAP), which escalate the primary vectors of the DENV by spreading *Aedes* mosquitoes [1, 3].

However, Bangladesh has been facing the re‐emerging dengue outbreak since the 2020 before the long‐term way of prevention of the disease. Now, it is now experiencing the most critical period of dengue outbreak in its history in terms of the number of cases and deaths. The Bangladesh government has taken many measures to control the outbreak, and hospitals are working to cope with a huge influx of patients beyond their capacity, and public health workers are focusing on controlling the mosquito populations through many activities such as vector control measures, public awareness campaigns and community engagement [4, 5]. Despite these efforts of measures, dengue outbreak is escalating rapidly with 102,861 dengue cases and 198 deaths in 2025 [6].

The dengue cases and deaths in Bangladesh have been re‐emerged as a public health issue that affects the mortality rate and life expectancy. Previous findings indicate the significant impact on gender‐ and age‐specific infection and mortality rates [7]. Dhaka city is one of the largest hotspots of dengue cases and deaths in Bangladesh, and other city corporations, such as Chattogram, Rajshahi, Rangpur, Mymensingh, Khulna, Barisal, and Sylhet, also have shown a higher concentration of dengue cases and mortality [8, 9]. In addition, the southeast and central regions of Bangladesh, especially urban areas and Dhaka city, exhibited the large number of dengue concentration than other regions [10]. Moreover, the increase of local temperature and changing rainfall season is one of the main factors for increasing dengue cases and deaths in Bangladesh [11, 12]. The peak incidence and mortality of dengue start from the earlier July to October, which affect almost 64 districts [10].

The age differences of population have an impact on the dengue incidence and mortality that are susceptible to the population at risk for dengue‐affected cases and deaths [13, 14]. Some attempts were made to previous studies to investigate age distribution differences of dengue incidence and mortality with case fatality in a year [7], but trends and patterns of dengue cases, deaths, and case fatality over the year from 2000 to 2025 are not addressed in Bangladesh. Despite susceptibility of age specific cases and mortality rates for dengue occur, most of the studies demonstrate demographic disparities, but not in significant trends of cases, deaths, and CFR. This study aims to assess the trends and patterns of incidence and mortality over the year from 2000 to 2025.

## 2 Methods

### 2.1 Data Sources and Collection

The data were collected from the Management Information System (MIS) of the Ministry of Health and Family Welfare, Bangladesh, which provide the daily reports of cases, deaths, and other information, and from the Institute of Epidemiology, Disease Control and Research (IEDCR), Bangladesh, from 01 January 2000 to 31 December 2025 [6, 15]. The two portals release the updated data by the government.

### 2.2 Data Processing and Statistical Analysis

The available secondary data were extracted from the portal, then cleaned, and coded using the Microsoft Excel. Descriptive analysis was also performed using the Microsoft Excel. The case fatality rate (CFR) was computed by dividing the total number of deaths by the total number of confirmed cases of dengue then multiplied by 100 to obtain a percentage. The monotonic trends in annual dengue cases, dengue deaths, and CFR in Bangladesh during 2000–2025 were assessed using the non‐parametric Mann–Kendall trend test that was used to determine the direction and strength of the monotonic trend with a positive value indicating an increasing trend, and a negative value indicating a decreasing trend. Statistical significance was assessed at the 5% significant level (*p* < 0.05). The magnitude of the trend was estimated using the Sen slope estimator with a 95% confidence interval (CI). The analyses were conducted in R (version 4.6.0) using RStudio. In addition, the Joinpoint regression software was used to analyze temporal trends in dengue cases and deaths and to estimate the annual percentage change (APC) with 95% CI.

### 2.3 Ethical Considerations

The government data are publicly available and do not need require formal ethical approval.

## 3 Results

### 3.1 Descriptive Analysis

In Bangladesh, dengue prevalence was much higher in 2000, but the trends became starting worst from 2015 and still the situation is becoming complex and alarming (Table 1). The trends of dengue cases, deaths, and CFRs in Bangladesh from 2000 to 2025 show substantial fluctuations, with notable increases in recent years. Between 2000 and 2010, the number of dengue cases remained relatively low. However, after 2010, a gradual increase became evident, particularly from 2018 onward. A sharp increase occurred in 2019 and reached around 101,354 cases that indicated a historic peak at that time. The highest reported dengue burden of cases was 321,179 and 1705 deaths in 2023, which was 1.3 times higher in occurred cases and 2.0 times higher in deaths than the total previous 22 years.

**TABLE 1:** Dengue trends in Bangladesh from 2000 to 2025.

Year	Cases	Deaths	Case fatality rate (%)	Year	Cases	Deaths	Case fatality rate (%)
**2000**	5551	93	1.68	**2014**	375	0	0.00
**2001**	2430	44	1.81	**2015**	3162	6	0.19
**2002**	6232	58	0.93	**2016**	6060	14	0.23
**2003**	486	10	2.06	**2017**	2769	8	0.29
**2004**	3934	13	0.33	**2018**	10,148	26	0.26
**2005**	1048	4	0.38	**2019**	101,354	164	0.16
**2006**	2200	11	0.50	**2020**	1405	7	0.50
**2007**	466	0	0.00	**2021**	28,429	105	0.37
**2008**	1151	0	0.00	**2022**	61,089	281	0.46
**2009**	472	0	0.00	**2023**	321,179	1705	0.53
**2010**	409	6	1.47	**2024**	101,214	575	0.57
**2011**	1359	1	0.07	**2025**	102,861	413	0.40
**2012**	641	2	0.31				
**2013**	1749	0	0.00				

Annual deaths showed also a similar fluctuating pattern but remained comparatively low in the early years. Between 2000 and 2018, mortality that ranged from 0 to 93 deaths annually with several years was reported very few or no deaths (Table 1). In 2019, 164 deaths were reported, rose sharply from 2022, and further peaked at 1705 deaths in 2023. In addition, the CFR varied over time in which highest was 2.06% in 2003. In the early 2000s, CFR was high, which fluctuated over time, but it began to increase again from 2020 (Table 1).

The number of dengue‐affected cases by age groups in Bangladesh showed some variation between 2023 and 2025 (Figure 1). The results show that the dengue disproportionately affected young‐ and middle‐aged populations. The majority of cases were concentrated among the aged 15–45 years, with a comparison with the children below 15 years and older population aged 60 years and above. The distribution of dengue‐related deaths across age groups followed a similar pattern to that of affected cases, but the concentration was greater among women and older age groups (Figure 2).

**FIGURE 1:**
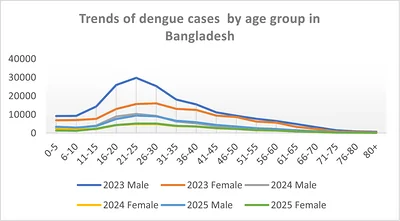
Trends of dengue‐affected cases by age group in Bangladesh from 2023 to 2025.

**FIGURE 2:**
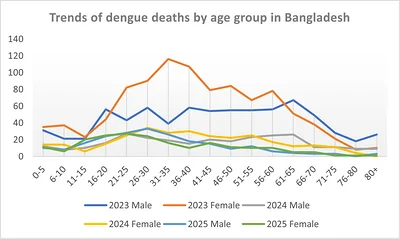
Trends of dengue death by age group in Bangladesh from 2023 to 2025.

In 2023, women had highest death than men in the history of Bangladesh. Although the working age population accounted for the majority of deaths in numbers, mortality rates were disproportionately higher among aged 60 years and above. This indicates that the greater vulnerability of elderly populations to severe dengue outcomes.

The CFR varied by age group. Although the CFR remained relatively low among children and working age population, it increased among older population, particularly those above 61 years of age, where the CFR values exceeded 1% (Figure 3). This finding suggests that although the working age population bears the greatest burden of cases and deaths, but older population remains the most vulnerable group in terms of dengue case fatal outcomes. The majority of the cases and deaths occurred from June to December, in which it peaked in August–October in each year (Figure 4).

**FIGURE 3:**
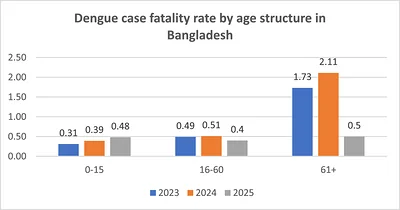
Dengue cases fatality rate by age structure in Bangladesh from 2023 to 2025.

**FIGURE 4:**
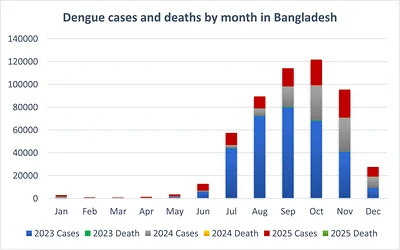
Dengue cases and deaths by months in Bangladesh from 2023 to 2025.

### 3.2 Trend Analysis of Dengue Over the Year

A significant increasing trend was observed in the annual dengue cases during the period of 2000–2025 (Mann–Kendall test statistic = 0.378, *p* = 0.007) (Table 2). Sen's slope estimate indicated an increase of 550.6 cases per year (95% CI: 84.82–2793.29). Dengue‐related deaths also demonstrated the increasing trend (Mann–Kendall test statistic = 0.257, *p* = 0.073), although the trend was not statistically significant with a Sen's slope of 1.25 deaths per year (95% CI: 0–9.00). In contrast, the CFR showed statistically not significant trend (Mann–Kendall test statistic = −0.066, *p* = 0.658) with a Sen's slope of −0.0046% per year (95% CI: −0.0408 to 0.0201).

**TABLE 2:** Trend analysis of annual dengue cases, deaths, and case fatality rate using the Mann–Kendall test and Sen's slope, 2000–2025.

Outcome	Mann–Kendall (M–K) test	*p* value	Sen's slope	Conclusion
Cases	0.378	0.007^*^	+550.6 (84.82–2793.29)	Significant increasing trend
Deaths	0.257	0.073	+1.25 (0–9.00)	Increasing but not significant
CFR	−0.066	0.658	−0.00462 (0.0408–0.0201)	No significant trend

Abbreviation: CI, confidence interval.

* *p* < 0.05.

### 3.3 Long‐Term Temporal Trend Analysis of Dengue

The Joinpoint regression identified a model with zero Joinpoint model as the best‐fitting model for annual reported dengue cases in Bangladesh during 2000–2025 (Table 3). The annual dengue case showed a significant increasing trend with an APC of 21.94% (95% CI: 18.95–42.75; *p* < 0.00001). The dengue‐related deaths also exhibited a statistically significant increasing trend with an APC of 15.09% (95% CI: 11.54–26.42; *p* < 0.001).

**TABLE 3:** Joinpoint regression analysis of annual dengue cases and deaths in Bangladesh, 2000–2025.

Outcome	APC (95% CI)	*p* value	Description
Cases	21.94 (18.95–42.75)	<0.001^*^	Significant increasing trend
Deaths	15.09 (11.54–26.42)	<0.001^*^	Significant increasing trend

Abbreviations: APC, annual percentage change; CI, confidence interval.

* *p* < 0.05.

## 4 Discussion

This study examined the trends and patterns of dengue cases, deaths, and CFRs over the years in Bangladesh from 2020 to 2025 as well as their distribution by age‐group and month from 2023 to 2025. The findings show a significant rising trend of dengue burden over the year. The data indicate that Bangladesh has entered a period of large‐scale dengue outbreak, departing from the modest transmission patterns observed before 2018. The dramatic increase occurred in 2023, when the country recorded more than 320,000 cases and over 1700 deaths, which represents the most severe outbreak in its history. The similar shifts have been observed in other dengue endemic regions of South Asia, Southeast Asia, Europe, and Latin America, where cyclical seasonal outbreaks have intensified in the over period [2, 16, 17, 18]. A study reveals that dengue cases and deaths were increased sharply by 6.5% and 11%, respectively, between 2000 and 2023 in South Asia where Bangladesh had higher CFR [19].

The significant increasing trend in reported dengue cases over the study period indicates a growing dengue burden in Bangladesh from 2020 to 2025. The similar study found in India in which dengue cases significantly increased over the year by using the same analysis of this study [20]. Another study in Mexico showed the long‐term significant increase of dengue burden [21]. But the study in West Bengal, India found the upward and downward trend of dengue burden over the year [22]. However, for the long‐term temporal trend of dengue in Bangladesh demonstrated that cases and deaths significantly increased over the year. A study found in South Asia context found the India, Pakistan, then Bangladesh marked as the highest burden of dengue cases than other countries [23]. A study in Indonesia described the dengue deaths a significantly increasing trend over the year, but not in dengue cases [24].

Although other factors, like rapid and unplanned urbanization, have led to poor waste management, inadequate drainage, and stagnant water bodies, all of which create favorable breeding habitats for the primary vector, *Aedes aegypti* [2, 17]. The incidence and death increase in the monsoon and post‐monsoon time of the month in Bangladesh, but not significantly. The findings aligned in the other South Asian countries and other regions [17, 19, 25]. Moreover, the CFR is relatively high in the Asia compared with other regions [2].

Moreover, several factors are contributed to a high number of incidences and deaths outside of Dhaka city, because the Dhaka's hospital treatment facilities are far better those in other region of Bangladesh. For this reason, people travel to Dhaka when are referred to for better treatment in well‐developed hospital management [8, 9, 11]. In addition, Dhaka city contains more than 10.3 million people, in which Dhaka division has more than 44 million of population density that is 2156/km^2^; thus people are exposed, and more than 80% of the people in Dhaka had antibodies against dengue [26, 27].

The age‐specific patterns of dengue incidence and mortality descriptively observed in this study are consistent with both global and regional literature. The majority of cases occurred among adolescents and working‐age populations aged between 15 and 45 years, reflecting high exposure levels among the working‐age population who are more likely to engage in outside works and live in densely populated urban areas. In contrast, children under 15 years and elderly are also accounted for the cases and death, though they remain a vulnerable group for severe manifestations due to immunological factors. The age group variation has also been observed in other regions in South Asia, Africa, and elsewhere [17, 18]. A study in southern Vietnam found that age specific cases, death, and CFR increase in adult and older age than younger age which opposite in recent dengue situation in Bangladesh [28].

The another study showed that the elderly population specially 60+ is at a higher risk of dengue than children [29]. Moreover, the elderly are at a high risk of severe dengue infection because of the more often age‐related co‐morbidities that can escalate dengue infection, especially among these, cardiovascular disease, diabetes, and respiratory disease are the most common [30]. This study shows that the dengue incidence and mortality rise from early July to end of the December, in which peak at August and September. Previous studies have shown the same pattern of dengue incidence and deaths [31, 32]. The study suggests the devastating conditions of dengue in Bangladesh that largely concentrate in Dhaka city and vary from age group, gender, and seasonal changes. In China, a study found that the prevalence of dengue is higher in 0–14 age children than others group that also has opposite results in Bangladesh [33]. This study has several notable limitations. The secondary surveillance data lacked of individual‐level information, potential underreporting, environmental, and socioeconomic variables. For this reason, it was not possible to establish causal relationship between variables. In addition, age‐ and sex‐specific data were only available from 2023 to 2025 that made the analysis more descriptive than analytical. Moreover, the study used the routinely collected government surveillance data, in which surveillance systems, diagnostic capacity, laboratory confirmation, or reporting practices over the 25‐year study period may change that could potentially affect the analysis of the trend.

## 5 Conclusion

This study found that significantly increasing trends and patterns of dengue over the year escalated the public health challenges in Bangladesh from 2000 to 2025, with large numbers of cases and deaths recorded over the period. Although CFRs have declined over time due to the improvements in clinical management, the incidence, mortality, and CFR indicated the vulnerability of the health system to seasonal epidemic surges. These study findings indicate that dengue cases and deaths showed a significantly long‐term rising trend that disproportionately affects the working‐age population, whereas older populations face greater risks of severe fatality outcomes and death. It suggests that more efforts are needed to government to control the exposure of dengue through effective programs. The significant increasing trend of dengue deaths suggests the ensuring government to attain health capacity for this re‐emerging outbreak. The persistence of dengue cases by older people, especially in Dhaka city, strong seasonal patterns drive in shaping of dengue transmission dynamics in Bangladesh with most of the dengue incidence and mortality occurring from July to December. The factors may contribute to dengue directly and indirectly need to be addressed through effective strategies by the government with considering urbanization and climate change.

## Author Contributions

Md. Idris Ali conceptualized, reviewed, edited, and finalized the manuscript.

## Funding

The author has nothing to report.

## Conflicts of Interest

The author declares no conflicts of interest.

## Data Availability

The data of this study were collected from the MIS, the Directorate General of Health Services, the Ministry of Health and Family Welfare, Bangladesh. Data are publicly available at the Daily Dengue Press Release (https://old.dghs.gov.bd/index.php/bd/home/5200‐daily‐dengue‐status‐report).

## References

[1] WHO , Dengue (WHO, 2025), https://www.who.int/news‐room/fact‐sheets/detail/dengue‐and‐severe‐dengue.

[2] N. Haider , M. N. Hasan , J. Onyango , et al., “Global Dengue Epidemic Worsens With Record 14 Million Cases and 9000 Deaths Reported in 2024,” International Journal of Infectious Diseases 158 (2025): 107940, 10.1016/j.ijid.2025.107940. 40449873

[3] Y. Araf , M. Akter , J. Zhai , C. Zheng , and M. G. Hossain , “Emerging Health Implications of Climate Change: Dengue Outbreaks and Beyond in Bangladesh,” Lancet Microbe 5, no. 3 (2024): e213, 10.1016/S2666-5247(23)00366-X. 38219754

[4] M. Asaduzzaman , E. A. Khan , M. N. Hasan , et al., “The 2023 Dengue Fatality in Bangladesh: Spatial and Demographic Insights,” IJID Regions 15 (2025): 100654, 10.1016/j.ijregi.2025.100654. PMC12136800 40469930

[5] M. M. Hasan , A. M. Sahito , M. Muzzamil , et al., “Devastating Dengue Outbreak Amidst COVID‐19 Pandemic in Bangladesh: An Alarming Situation,” Tropical Medicine and Health 50, no. 1 (2022): 11, 10.1186/s41182-022-00401-y. PMC8786620 35078540

[6] DGHS , Daily Dengue Press Releases (DGHS, 2025), https://old.dghs.gov.bd/index.php/bd/home/5200‐daily‐dengue‐status‐report.

[7] S. Ashraf , M. M. Patwary , and A. J. Rodriguez‐Morales , “Demographic Disparities in Incidence and Mortality Rates of Current Dengue Outbreak in Bangladesh,” New Microbes and New Infections 56 (2023): 101207, 10.1016/j.nmni.2023.101207. PMC10746555 38143942

[8] N. Haider , M. Asaduzzaman , M. N. Hasan , et al., “Bangladesh's 2023 Dengue Outbreak—Age/Gender‐Related Disparity in Morbidity and Mortality and Geographic Variability of Epidemic Burdens,” International Journal of Infectious Diseases 136 (2023): 1–4, 10.1016/j.ijid.2023.08.026. 37660728

[9] M. N. Hasan , M. Rahman , M. Uddin , et al., “The 2023 Fatal Dengue Outbreak in Bangladesh Highlights a Paradigm Shift of Geographical Distribution of Cases,” Epidemiology and Infection 153 (2025): e3, 10.1017/S0950268824001791. PMC11704938 39763239

[10] K. Hossain , S. Chowdhury , I. S. Shanta , M. S. Hossain , P. K. Ghosh , and M. S. Alam , “Spatio‐Temporal Patterns of Dengue in Bangladesh During 2019 to 2023: Implications for Targeted Control Strategies,” PLoS Neglected Tropical Diseases 18, no. 9 (2024): e0012503, 10.1371/journal.pntd.0012503. PMC11446421 39302980

[11] M. N. Hasan , I. Khalil , M. A. B. Chowdhury , et al., “Two Decades of Endemic Dengue in Bangladesh (2000–2022): Trends, Seasonality, and Impact of Temperature and Rainfall Patterns on Transmission Dynamics,” Journal of Medical Entomology 61, no. 2 (2024): 345–353, 10.1093/jme/tjae001. PMC10936167 38253990

[12] O. Titus Muurlink , P. Stephenson , M. Z. Islam , and A. W. Taylor‐Robinson , “Long‐Term Predictors of Dengue Outbreaks in Bangladesh: A Data Mining Approach,” Infectious Disease Modelling 3 (2018): 322–330, 10.1016/j.idm.2018.11.004. PMC6326231 30839927

[13] E. Annan , J. Treviño , B. Zhao , A. J. Rodriguez‐Morales , and U. Haque , “Direct and Indirect Effects of Age on Dengue Severity: The Mediating Role of Secondary Infection,” PLoS Neglected Tropical Diseases 17, no. 8 (2023): e0011537, 10.1371/journal.pntd.0011537. PMC10441797 37556473

[14] T. Zohra , M. Din , A. Ikram , et al., “Demographic and Clinical Features of Dengue Fever Infection in Pakistan: A Cross‐Sectional Epidemiological Study,” Tropical Diseases, Travel Medicine and Vaccines 10, no. 1 (2024): 11, 10.1186/s40794-024-00221-4. PMC10998367 38581059

[15] IEDCR , Dengue Situation Update (IEDCR, 2023), https://www.iedcr.gov.bd/site/page/54072db2‐9b03‐4481‐b88a‐adaa47e6d8d7/http%3A%2F%2Fwww.iedcr.gov.bd%2Fsite%2Fpage%2F54072db2‐9b03‐4481‐b88a‐adaa47e6d8d7%2F‐.

[16] European Centre for Disease Prevention and Control , Dengue Worldwide Overview (European Centre for Disease Prevention and Control, 2025), https://www.ecdc.europa.eu/en/dengue‐monthly.

[17] X. Yang , M. B. M. Quam , T. Zhang , and S. Sang , “Global Burden for Dengue and the Evolving Pattern in the Past 30 Years,” Journal of Travel Medicine 28, no. 8 (2021): taab146, 10.1093/jtm/taab146. 34510205

[18] S. X. Zhang , G. B. Yang , R. J. Zhang , et al., “Global, Regional, and National Burden of Dengue, 1990–2021: Findings From the Global Burden of Disease Study 2021,” Decoding Infection and Transmission 2 (2024): 100021, 10.1016/j.dcit.2024.100021.

[19] M. Asaduzzaman , M. N. Hasan , M. A. O. Nasif , et al., “Unravelling the Dengue Surge in South Asia During 2000–2023: Pattern, Trend, Genomics, and Key Determinants,” Epidemiology and Infection 154 (2026): e28, 10.1017/S0950268826101095. PMC12964148 41644307

[20] B. Mandal and S. Mondal , “Unveiling Spatio‐Temporal Mysteries: A Quest to Decode India's Dengue and Malaria Trend (2003–2022),” Spatial and Spatio‐Temporal Epidemiology 51 (2024): 100690, 10.1016/j.sste.2024.100690. 39615969

[21] J. Briseno‐Ramirez , J. C. De Arcos‐Jiménez , A. M. López‐Yáñez , et al., “Trends and Cyclical Patterns of Dengue Disease in Mexico: A 40‐Year Time Series Analysis,” Annals of Epidemiology 112 (2025): 53–63, 10.1016/j.annepidem.2025.10.022. 41187838

[22] J. Majhi , R. Singh , V. Yadav , et al., “Dynamics of Dengue Outbreaks in Gangetic West Bengal: A Trend and Time Series Analysis,” Journal of Family Medicine and Primary Care 9, no. 11 (2020): 5622–5628, 10.4103/jfmpc.jfmpc_800_20. PMC7842458 33532405

[23] R. Velyutham , S. Kalra , S. Yadav , et al., “Trends in Dengue Incidence and Disease Burden in South Asia With Special Reference to India: Insights From the Global Burden of Disease Data, 1990–2021,” Journal of Infection and Public Health 19 (2026): 103142. 10.1016/j.jiph.2026.103142 41666533

[24] A. Sudaryanto , W. C. Liao , Y. P. Lin , and W. C. Ho , “Two Decades of Dengue in Indonesia: Long‐Term Trends in Incidence, Mortality, and Disability‐Adjusted Life Years, 2005–2024,” Pathogens 15, no. 4 (2026): 373, 10.3390/pathogens15040373. PMC13119181 42075700

[25] P. Mutsuddy , S. Tahmina Jhora , A. K. M. Shamsuzzaman , S. M. G. Kaisar , and M. N. A. Khan , “Dengue Situation in Bangladesh: An Epidemiological Shift in Terms of Morbidity and Mortality,” Canadian Journal of Infectious Diseases Medical Microbiology 2019, no. 1 (2019): 3516284, 10.1155/2019/3516284. PMC6431455 30962860

[26] P. Dhar‐Chowdhury , K. K. Paul , C. E. Haque , et al., “Dengue Seroprevalence, Seroconversion and Risk Factors in Dhaka, Bangladesh,” PLoS Neglected Tropical Diseases 11, no. 3 (2017): e0005475, 10.1371/journal.pntd.0005475. PMC5380355 28333935

[27] Bangladesh Bureau of Statistics (BBS) , Bangladesh Population and Housing Census 2022 Bangladesh Bureau of Statistics, and Information Division, Ministry of Planning (Bangladesh Bureau of Statistics (BBS), 2023), https://bbs.portal.gov.bd/sites/default/files/files/bbs.portal.gov.bd/page/b343a8b4_956b_45ca_872f_4cf9b2f1a6e0/2024‐01‐31‐15‐51‐b53c55dd692233ae401ba013060b9cbb.pdf.

[28] A. F. Taurel , C. Q. Luong , T. T. T. Nguyen , et al., “Age Distribution of Dengue Cases in Southern Vietnam From 2000 to 2015,” PLoS Neglected Tropical Diseases 17, no. 2 (2023): e0011137, 10.1371/journal.pntd.0011137. PMC9994699 36827445

[29] E. K. Rowe , Y. S. Leo , J. G. X. Wong , et al., “Challenges in Dengue Fever in the Elderly: Atypical Presentation and Risk of Severe Dengue and Hospital‐Acquired Infection,” PLoS Neglected Tropical Diseases 8, no. 4 (2014): e2777, 10.1371/journal.pntd.0002777. PMC3974675 24699282

[30] J. Toledo , L. George , E. Martinez , et al., “Relevance of Non‐Communicable Comorbidities for the Development of the Severe Forms of Dengue: A Systematic Literature Review,” PLoS Neglected Tropical Diseases 10, no. 1 (2016): e0004284, 10.1371/journal.pntd.0004284. PMC4699776 26727113

[31] A. Ahsan , N. Haider , R. Kock , and C. Benfield , “Possible Drivers of the 2019 Dengue Outbreak in Bangladesh: The Need for a Robust Community‐Level Surveillance System,” Journal of Medical Entomology 58 (2020): 37–39, 10.1093/jme/tjaa150. 32725192

[32] K. K. Bhowmik , J. Ferdous , P. K. Baral , and M. S. Islam , “Recent Outbreak of Dengue in Bangladesh: A Threat to Public Health,” Health Science Reports 6, no. 4 (2023): e1210, 10.1002/hsr2.1210. PMC10090488 37064322

[33] L. Tao , Y. Zou , T. Liu , et al., “Trends in Burdens of Dengue Fever Among Children Aged 0‒14 Years in China From 1990 to 2021: Findings From the Global Burden of Disease Study 2021, and Projections Up to 2035,” Global Health Journal 9, no. 2 (2025): 145–152, 10.1016/j.glohj.2025.06.010.

